# Supplementary material for: Discrepancies between observations and climate models of large-scale wind-driven Greenland melt influence sea-level rise projections
Source: Nat Commun. 2022 Nov 14;13:6833. doi: 10.1038/s41467-022-34414-2 (PMC9663692; doi:10.1038/s41467-022-34414-2)
Supplement: Supplementary file 1 — Supplementary Information [file 41467_2022_34414_MOESM1_ESM.pdf]

SUPPLEMENTARY INFORMATION FOR

Discrepancies between observations and climate models of large-scale wind-driven Greenland melt influence sea-level rise projections

Dániel Topál[1,2,3*], Qinghua Ding[2*], Thomas J. Ballinger[4*], Edward Hanna[5], Xavier Fettweis[6], Zhe Li[2] & Ildikó Pieczka[7]

[1]Institute for Geological and Geochemical Research, Research Centre for Astronomy and Earth Sciences, MTA-Centre for Excellence, ELKH, 45 Budaörsi Street, H-1112, Budapest, Hungary

[2]Department of Geography and Earth Research Institute, University of California-Santa Barbara, Santa Barbara, CA, USA

[3]ELTE Eötvös Loránd University, Doctoral School of Environmental Sciences, Budapest, Hungary

[4]International Arctic Research Center, University of Alaska Fairbanks, Fairbanks, AK, USA

[5]School of Geography and Lincoln Climate Research Group, University of Lincoln, Lincoln, United Kingdom

[6]SPHERES research units, Geography Department, University of Liège, Liège, Belgium

[7]ELTE Eötvös Loránd University, Institute of Geography and Earth Sciences, Department of Meteorology, Budapest, Hungary

*Corresponding authors: topal.daniel@csfk.org; qinghua@ucsb.edu; tjballinger@alaska.edu

SUPPLEMENTARY DISCUSSION

Using MCA to reveal tropical drivers of Greenland atmospheric circulation changes

By applying maximum covariance analysis (MCA) to the Northern Hemisphere (NH) Z500 and tropical-to-midlatitude (-30ºS to 55ºN) SST fields (first removing the corresponding global (60ºS to 60ºN SST) area-weighted average value from the ERA5 Z500 and ERSSTv5 in each year and at each grid cell, respectively, to retain just internal variability), we reveal the leading internal coupled mode of variability between circulation and SSTs in both ERA5 and the EKF400 products. Note that how the estimated anthropogenic forcing is removed from observations (e.g., removing the linear or quadratic trends instead of the global mean) does not influence the results. Having done so, we exclude the use of any arbitrary predetermined circulation modes during our analysis, such as the North Atlantic Oscillation, that are often used to explain observed GrIS surface conditions. This is in line with our aim to show how wind-driven circulation coupled with SST-regulated Greenland surface conditions vary consistently over the past 400 years. Since the seasonality of tropical-Arctic teleconnections are not yet fully understood, we use annual means when placing the observed local GrIS circulation variability into hemispheric context, which enables a direct comparison with paleoclimatic proxy data assimilated model experiments and ice core records and also alleviates current uncertainties surrounding tropical-Arctic teleconnections.

Uncertainties around quantifying tropical teleconnections' role in GrIS surface changes

The leading internal MCA mode is also moderately and significantly correlated with the summer GSI from ERA5, which suggests that fingerprints of this teleconnection can be found in the summer season ($r_{Z500;GSI}$ = 0.56 (0.47); $r_{SST;GSI}$ = 0.47 (0.36), with detrended values in brackets), despite known uncertainties imposed by the summer monsoon, which affects its stability[64]. Based on the correlations between the GSI and the global SST/Z500 patterns (Supplementary Fig. 9) we speculate that the tropically-driven part of the local GSI changes might be up to 25% in annual means and about 15% in summer; however, these estimates are limited by inadequate representation of tropical SST variability in climate models. We note, that the use of available Pacific pacemaker experiments with CMIP5/6 climate models would provide minimal added insight since those experiments are forced by both specified SST (or nudged SST) and anthropogenic forcing. Currently there are no available pacemaker control simulations with fixed anthropogenic forcing, thus we would not be able to isolate the response of the model to imposed tropical SST forcing only. Additionally, based on previous calculations by the authors both CESM1 and CESM2 models have limilatitions in simulating the observed tropical rainfall trends in the Pacific, which is essential to the formation of tropical-Arctic teleconnections.

On the robustness of the MCA pattern seen in ERA5

We repeat the MCA calculations using the extended ERA5 reanalysis for the period 1950-2018. This analysis showed very similar spatial patterns to those seen in Fig. 4 in the main text and the expansion coefficient time series of the MCA as well as strong temporal agreement based on the extent to which the extended reanalysis and the shorter (1980-2018) period are correlated >0.95. This suggests that the PARC mode is a robust mode of atmosphere-ocean coupled variability. We note that the expansion coefficient time series from the extended ERA5 reanalysis also are highly correlated with the expansion coefficients derived from the MCA on the EKF400 database (~0.7).

Nudging run is robust in annual means

In the main text we have focused on the melt season (summer). However, we estimate that ~52% of the annual mean MAR GrIS SAT trends between 1990-2012 may be attributable to changes in the nudging experiment derived annual mean GSI with a similar spatial pattern to both our summer estimates and to the year-to-year regression of the GSI onto GrIS SAT or SMB using annual means (Supplementary Fig. 5d,e). Also, the year-to-year correlation between the GSI and both the EBAF summer (Supplementary Fig. 4a) and annual mean SEB (Supplementary Fig. 4c) show marked spatial patterns with significant positive correlations

over the Baffin Bay and in west Greenland in agreement with regions showing the most significant SMB and SAT changes since 1980 (Supplementary Fig. 3b-d).

Insensitivity of the linear trends to the time period

Regarding the sensitivity of our results based on linear trends to the time period chosen, we note that using 1980-2018 or 1980-2019/2020 did not affect our conclusions that are based on the 1990-2012 period. The nudging experiment explains a similar amount (~50%) of the observed Greenland surface air temperature trends during 1980-2018, 1990-2012 or 1980-2019/2020. Furthermore, as seen in Fig. 1 in the main text, the nudging experiment derived Greenland SAT or SMB in Fig. 2f both well resembles the observed 2012 high- and 2013 low-melt years, which indicates that the wind-driven mechanisms is at play at both interannual and decadal time scales.

LMR2.1 to verify EKF400 results

To further assess the robustness of the observed and EKF400 simulated teleconnection to impact GrIS warming over centennial time scales we also use the Last Millennium Reanalysis 2[55] proxy-assimilated reanalysis. The correlations between the GSI derived from the EKF400 (using 200hPa horizontal winds as per data availability) and the global mean removed Z500 and SST spatial patterns in both the EKF400 (over AD 1602-2003) and LMR2.1 (over AD 1602-2000) indicate, that while the EKF400 (Supplementary Fig. 11a-b) shows promising similarity to the correlations calculated between the ERA5 GSI and the reanalysis SST/Z500 (Supplementary Fig. 9) regarding both magnitudes and spatial patterns, the LMR2.1 exhibits smaller correlation magnitudes (Supplementary Fig. 11c-d) and less resemblance to the PARC. As for the MCA neither of the first two LMR2.1 MCA modes show adequate similarity to the PARC teleconnection, unlike the EKF400 which remarkably resembles the MCA seen in the ERA5 reanalysis (see Fig. 5 in the main text). However, the regression of the Z500 and SST fields from the LMR2.1 onto the Z500/SST expansion coefficient time series obtained from the EKF400, (after removing the global mean time-series from LMR2.1) still resemble the features of both the observed and the EKF400 simulated hemispheric teleconnections, which indicates that the PARC mode exists in the LMR2.1, but is not as separable from other modes as in the EKF400 (Supplementary Fig. 12).

SUPPLEMENTARY TABLES

Supplementary Table 1. 30 ice core records across the GrIS used in the study and the correlation coefficients between the individual ice cores and the MCA(1) related expansion coefficient time-series of Z500 (r<Z500>) and the 200hPa Greenland streamfunction index (r<Ψ200>) in the EKF400 simulation. The source codes of the oceanic coral records are also shown in the rightmost column referring to the codes of the original datasets in ref.[75]. The bold coefficients mark significant correlations ($p<0.05$).

**Ice Core Site**	**Lat**	**Lon**	**r(Z500)**	**r(Ψ200)**	**Coral proxy site[75]**
Prince-of-Wales	78.4	-80.4	-0.13	-0.24	Ocn_095
B26	77.25	-49.22	-0.05	-0.09	Ocn_111
B19	78	-36.4	-0.03	-0.01	Ocn_087
B27	76.66	-46.48	0.06	0.02	Ocn_140
B21	80	-41.14	-0.06	0.02	Ocn_073
B22	79.34	-45.91	-0.01	0.03	Ocn_099
Agassiz A79	80.7	-73.1	0.03	0.05	Ocn_078
Camp Century	77.17	-61.13	-0.08	0.1	Ocn_156
B16	73.94	-37.63	-0.07	0.1	Ocn_109
B20	78.83	-36.5	-0.08	0.1	Ocn_110
GRIP	72.58	-37.64	0.15	0.1	Ocn_060
Site G	71.76	-35.85	0.05	0.1	Ocn_122
Devon Ice Cap	75.33	-82.5	-0.08	0.12	Ocn_097
B18	76.62	-36.4	-0.02	0.16	Ocn_090
B30	75	-42	0.04	0.16	Ocn_081
Site E	71.15	-35.84	0.12	**0.18**	Ocn_080
B23	78	-44	0.19	**0.19**	Ocn_120
Agassiz A87	80.7	-73.1	-0.04	**0.19**	Ocn_062
B17	75.25	-37.62	0.11	**0.2**	Ocn_098
B28	76.66	-46.48	0.13	**0.2**	Ocn_075
Renland	71.27	-26.73	0.11	**0.21**	Ocn_125
NEEM	77.45	-51.06	0.17	**0.22**	Ocn_130
B29	76	-43.49	0.09	**0.22**	Ocn_103
Site A	70.63	-35.82	0.14	**0.23**	Ocn_179
GISP2	72.6	-38.5	0.16	**0.25**	Ocn_068
Site D	70.64	-39.62	**0.2**	**0.25**	Ocn_084
Crete	71.12	-37.32	0.14	**0.28**	Ocn_079
DYE2	66.38	-46.18	0.16	**0.29**	Ocn_083
NGRIP	75.1	-42.32	**0.26**	**0.3**	Ocn_077
Milcent	70.3	-44.58	**0.28**	**0.38**	Ocn_074
					Ocn_088
					Ocn_119
					Ocn_086

Supplementary Table 2. 31 climate models in the CMIP5 historical+RCP8.5 (1980-2018) experiment that were used in the study.

CMIP5 model name
1. ACCESS1-0
2. ACCESS1-3
3. CanESM2
4. CMCC-CESM
5. CMCC-CM
6. CMCC-CMS
7. CNRM-CM52
8. CNRM-CM5
9. CSIRO-Mk3.6.0
10. GFDL-CM3
11. GFDL-ESM2G
12. GFDL-ESM2M
13. GISS-E2-H
14. GISS-E2-H-CC
15. GISS-E2-R
16. GISS-E2-R-CC
17. HadGEM2-CC
18. HadGEM2-ES
19. INM-CM4
20. IPSL-CM5A-LR
21. IPSL-CM5B-LR
22. IPSL-CM5A-MR
23. MIROC-ESM
24. MIROC-ESM-CHEM
25. MIROC5
26. MPI-ESM-LR
27. MPI-ESM-MR
28. MPI-ESM-P
29. MRI-CGCM3
30. NorESM1-M
31. NorESM1-ME

SUPPLEMENTARY FIGURES

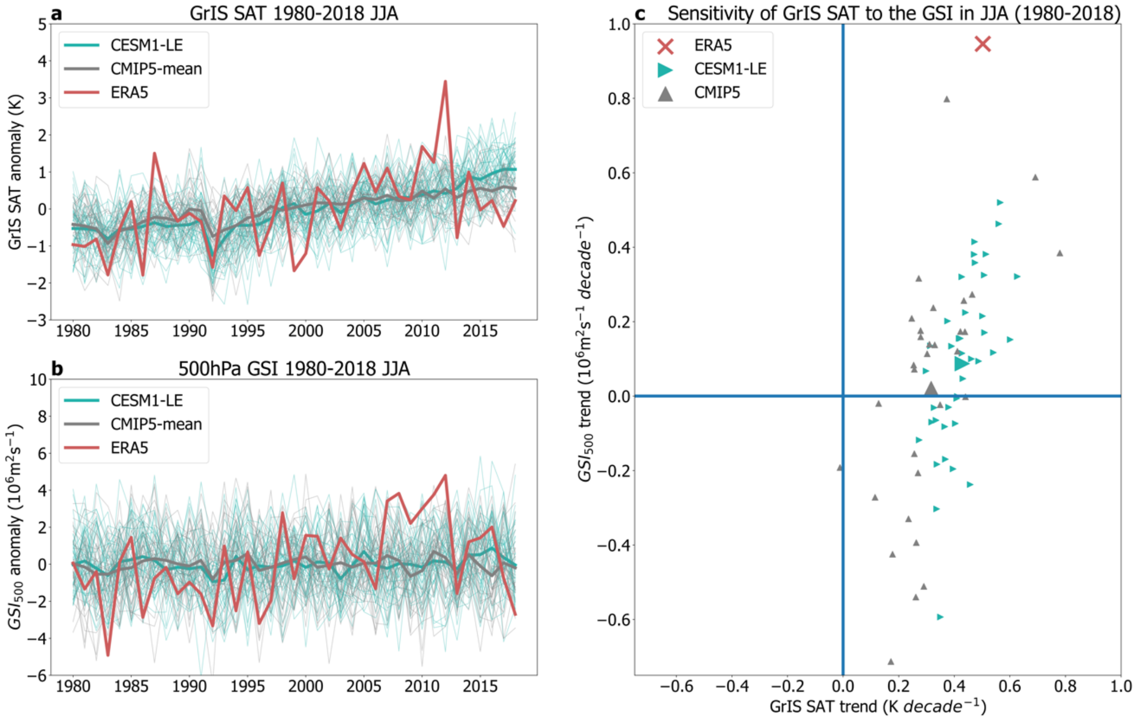

Supplementary Figure 1. (a) Greenland ice sheet (GrIS) surface air temperatures (SAT) in ERA5, the 40 member CESM1-LE (ensemble mean with thick light blue line) and in 31 CMIP5 models (multi-model ensemble mean with thick grey line) between 1980-2018 in June-July-August. (JJA). (b) the same for the Greenland Streamfunction Index calculated from 500hPa horizontal winds, and (c) scatter plot of the trends in the GSI and the GrIS SAT in each member of the CESM1-LE (seagreen triangles; ensemble mean with larger marker), each CMIP5 model (grey markers; ensemble mean with larger marker) and in ERA5 (red 'x'). Note how each CMIP5 and CESM1-LE members simulate GrIS warming without concomitant changes in their simulated GSI.

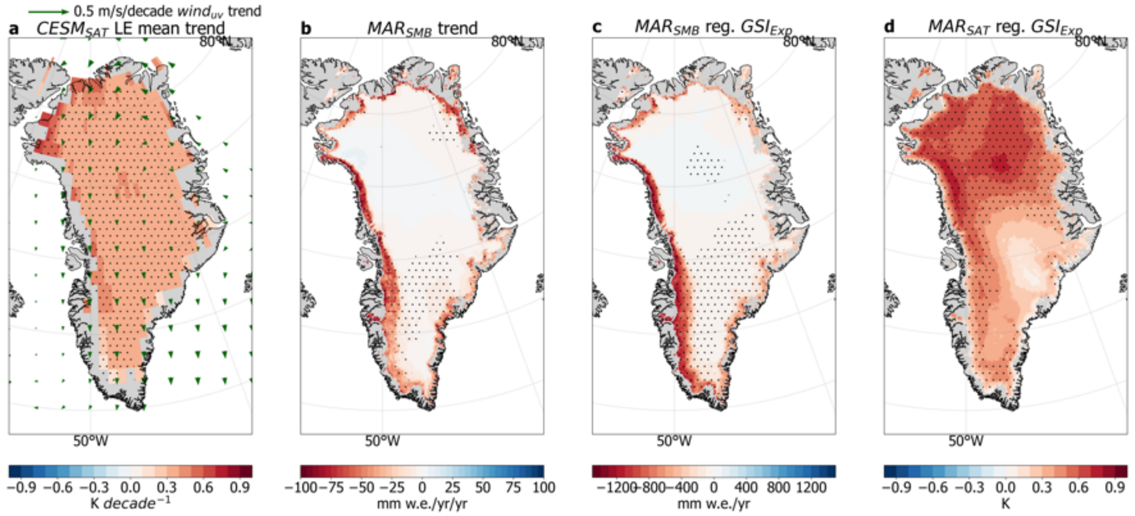

Supplementary Figure 2. Spatial patterns of the linear trends in (a) the 40-member CESM1 large ensemble mean surface air temperature (SAT) and 500hPa horizontal wind in addition to (b) the MAR surface mass balance (SMB) in summer (June-July-August). Also shown: maps of the MAR (c) SMB and (d) SAT regressions onto the 500hPa Greenland streamfunction index (GSI) derived from the nudging experiment.

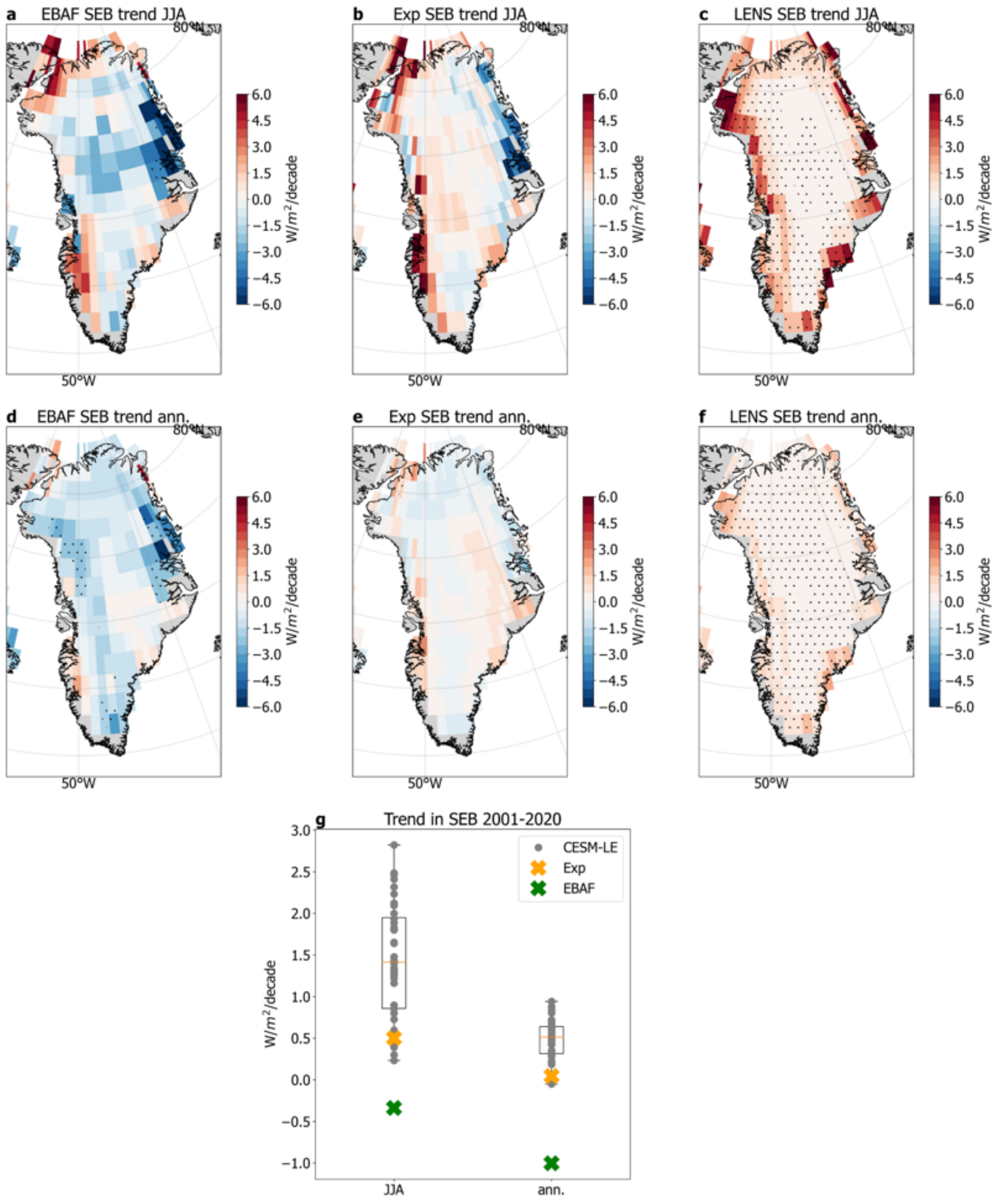

Supplementary Figure 3. Maps of the linear trends in the surface energy balance (SEB = SWnet+LWnet) in (a) the EBAF satellite product, (b) the nudging experiment (Exp) and (c) the CESM1 large ensemble mean (LENS) in summer (June-July-August, JJA) during 2001-2020. (d)-(f) is the same as (a)-(c) but for annual means. Panel (g) shows the GrIS spatially averaged linear trends in the SEB from the 40-member CESM1 large ensemble (grey dots) and the nudging experiment (Exp, orange marker) along with the EBAF satellite product (green marker) in JJA (first column) and annual means (second column) for 2001-2020.

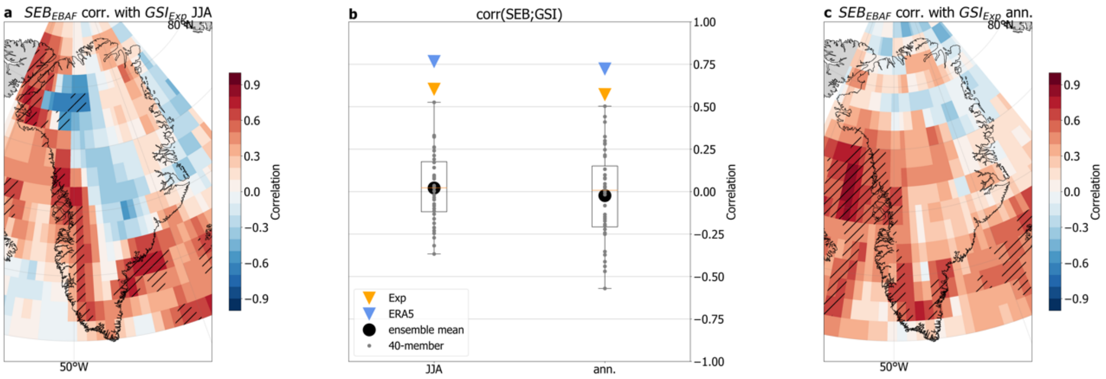

Supplementary Figure 4. Panels (a) and (c) show the correlation between EBAF SEB and the nudging experiment derived 500hPa Greenland streamfunction index (GSI) for JJA and annual means, respectively, and (b) shows the correlation between EBAF SEB and GSI in ERA5/the nudging experiment along with the correlation between SEB and GSI in each member of the CESM-LE and the mean of each member's correlation for JJA (left box plot) and annual means (right box plot) (see legend in panel (b)). Hatching in (a),(c) indicate areas with statistically-significant correlations ($p<0.05$).

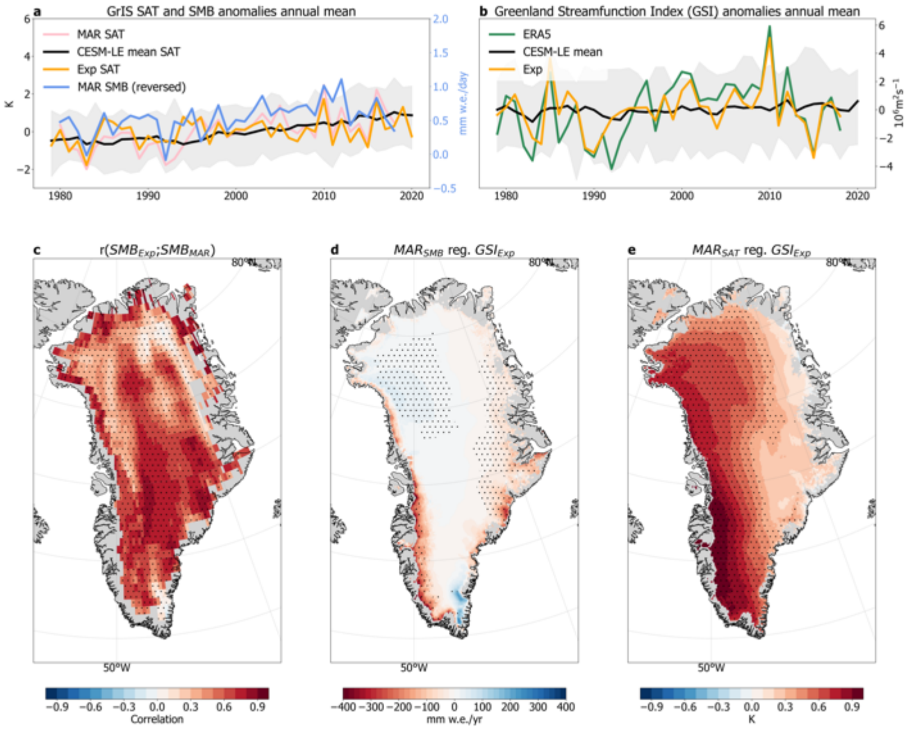

Supplementary Figure 5. (a) GrIS spatially-averaged time-series of anomalies in MAR surface air temperature (SAT, pink) and surface mass balance (SMB, blue), the ensemble mean SAT from CESM1 large ensemble (CESM-LE, black) and the SAT from the wind-nudging experiment (Exp) in CESM1 (orange) for the annual means. The grey shading in (a) represents all members' SAT anomalies from the CESM-LE. In (b) ERA5 500hPa Greenland streamfunction index (GSI) (green) is compared with the GSI calculated using the CESM-LE mean (black), the spread in CESM-LE (grey shading) and the wind-nudging experiment (Exp, orange) for annual means. Also shown: (c) the grid-point-wise correlation between the MAR and the CISM Glimmer experiment derived annual mean SMB along with the regression maps of (d) MAR SMB and (e) MAR SAT onto the 500hPa GSI derived from the nudging experiment. Hatching in (c)-(e) indicate areas with statistically-significant correlations or regressions ($p<0.05$).

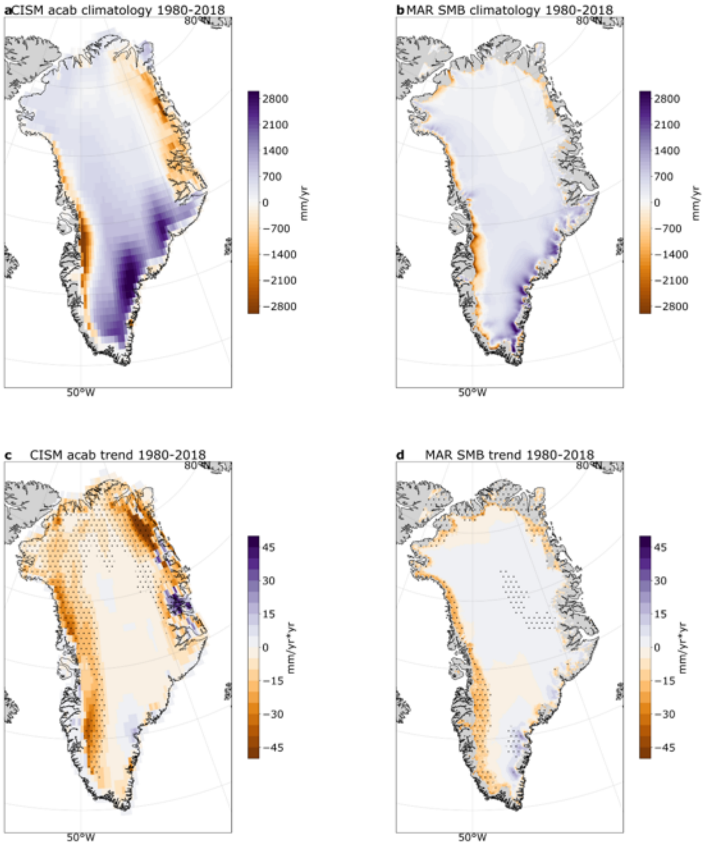

Supplementary Figure 6. Comparison between the (a) nudging experiment-driven CISM Glimmer simulation and the (b) MAR SMB (forced by ERA5) spatial climatologies for 1980-2018 annual means along with the linear SMB trend maps in the (c) Glimmer simulation and in (d) MAR.

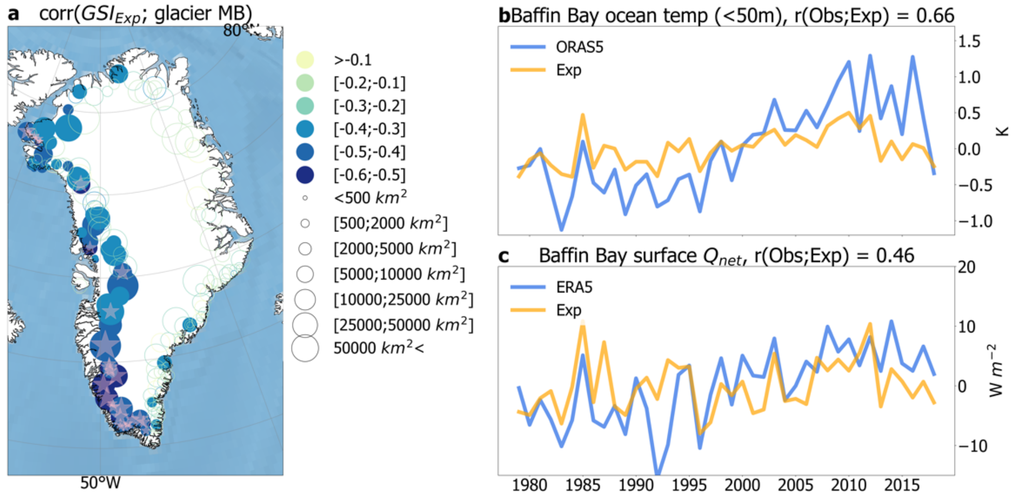

Supplementary Figure 7. In (a) the correlations between the summer GSI and the annual mean rate of mass balance of 260 glaciers from ref.[46] during 1980-2018 are shown. Filled circles indicate statistically-significant correlations ($p<0.05$) and pink stars denote those glaciers that show significant correlations even after detrended. The size of the markers are proportional to the area of the given glacier (see legend). Also shown: (b) JJA upper-ocean (0 – 50 m) temperatures and (c) surface net heat flux from the atmopshere into the ocean spatially averaged over 50-70°W 60-77°N between 1990-2012 in ORAS5/ERA5 reanalysis (blue) and in the nudging experiment (Exp, gold).

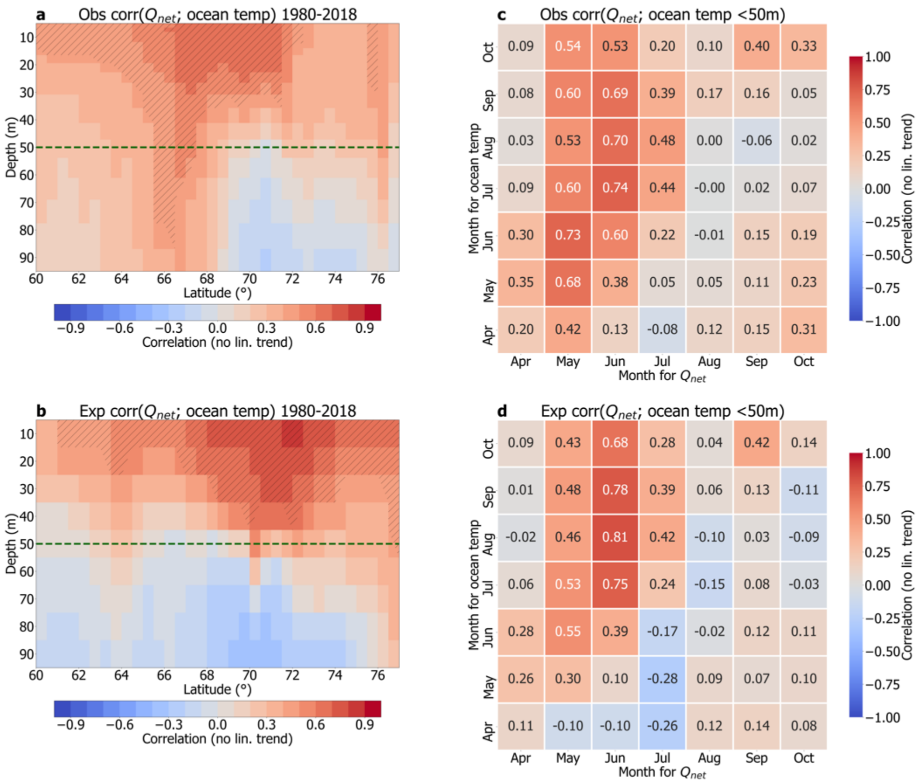

Supplementary Figure 8. Correlations between the net surface heat flux from the atmosphere into the ocean (Q_{net}) averaged over the Baffin Bay (50-70ºW; 60-77ºN) and upper ocean temperature (zonal mean over 50ºW-70ºW longitudinal band) in (a) the ORAS5 reanalysis and (b) in the nudging experiment over 1980-2018. Hatcing indicates statistically significant trends ($p<0.05$). Also shown: lead-lag correlation between the net surface heat flux from the atmosphere into the ocean (Q_{net}) averaged over the Baffin Bay (50-70ºW; 60-77ºN) and the upper 50m averaged ocean temperature (zonal mean over 50ºW-70ºW longitudinal band) in (c) ERA5/ORAS5 and (d) in the nudging experiment from April to October between 1980-2018. The linear trends are removed before correlations are calculated.

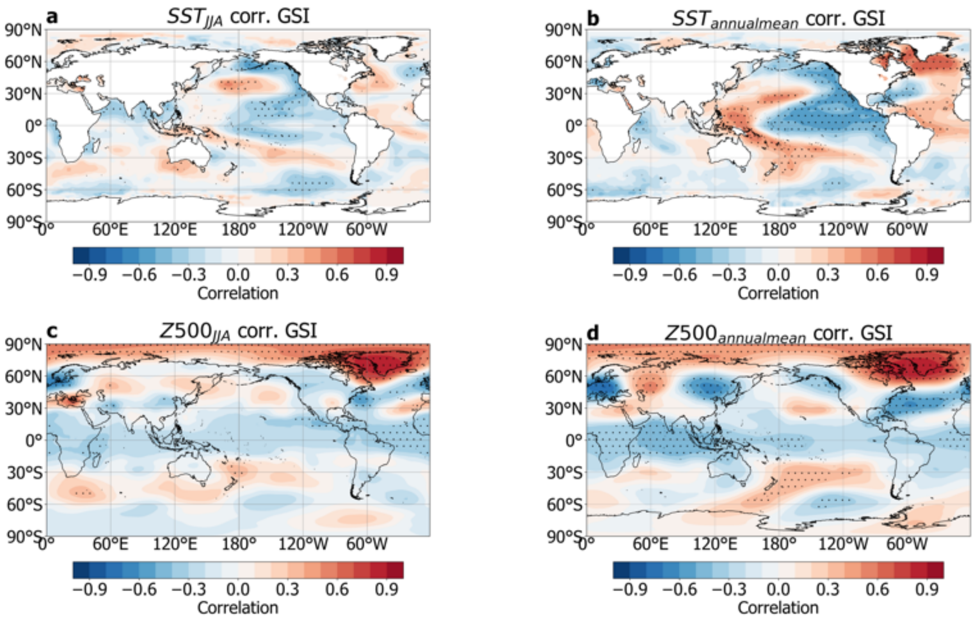

Supplementary Figure 9. Correlation between detrended (a)-(b) ERSSTv5 sea surface temperatures (SST) and (c)-(d) detrended 500hPa Greenland streamfunction index calculated from ERA5 (GSI, Methods) using (a),(c) summer-mean (June-July-August, JJA) and in (b),(d) using annual-mean fields for the period of 1980-2018. Hatching indicates statistically-significant correlations ($p<0.05$).

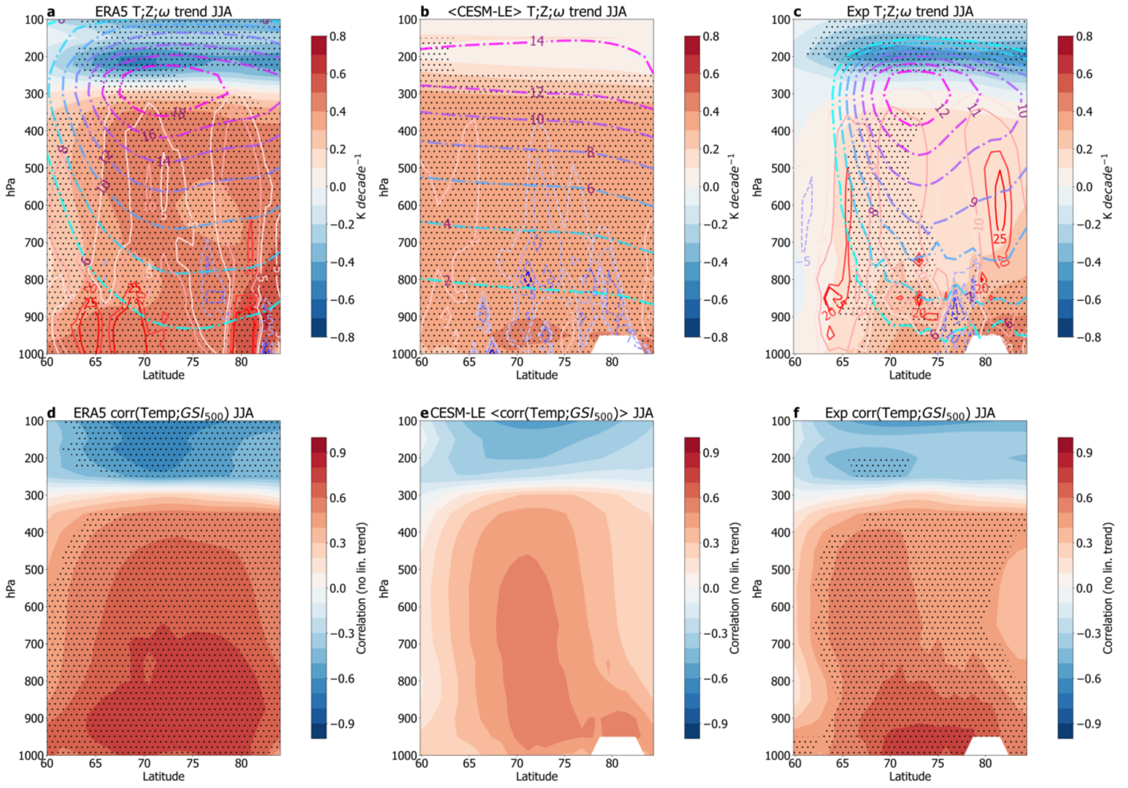

Supplementary Figure 10. Linear trend in zonal-mean temperature (shading), zonal-mean geopotential height (dash-dot contours; unit: m/decade) and vertical motion (omega; solid contours; unit: 10^5 Pa/s) over the GrIS (59ºN-85ºN;80ºW-20ºW) in (a) ERA5, (b) the mean of 40-members (forced component) of the CESM-LE and (c) nudging experiment between 1980-2018 JJA. Correlation between detrended zonal-mean temperature over the GrIS and 500hPa Greenland Streamfunction Index (GSI) in (d) ERA5, (e) the mean of the 40 individual correlation maps of the CESM-LE and (f) in the nudging experiment between 1980-2018 June-August (JJA).

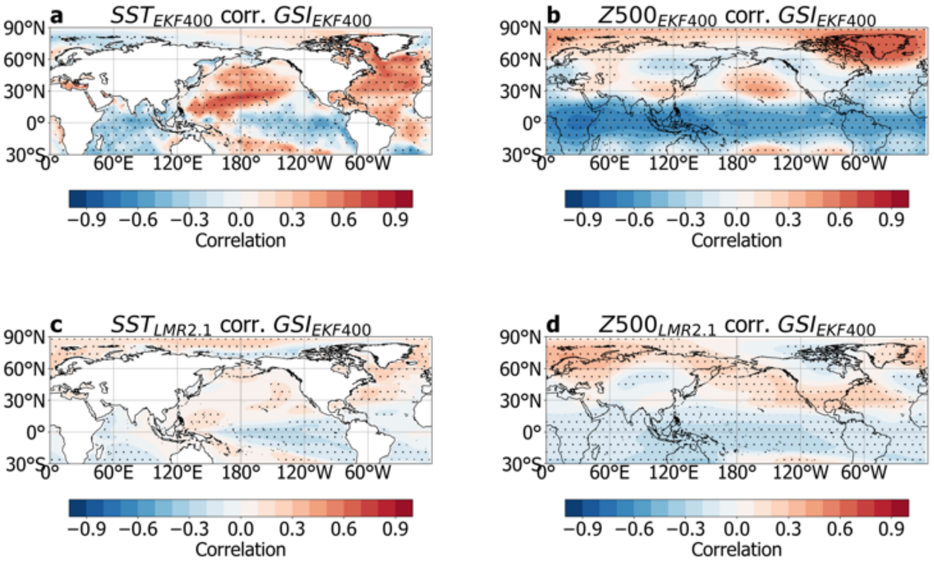

Supplementary Figure 11. Correlation between annual mean 200hPa Greenland streamfunction index (GSI, Methods) from the EKF400 and (a) sea surface temperatures (SST) in the EKF400 paleo-reanalysis, (b) 500hPa geopotential heights (Z500) from the EKF400 paleo-reanalysis, (c) SST from the LMR2.1 paleo-reanalysis, (d) Z500 from the LMR2.1 paleo-reanalysis for the period of AD 1602-2003 using annual mean fields. Hatching indicates statistically significant correlations ($p<0.05$).

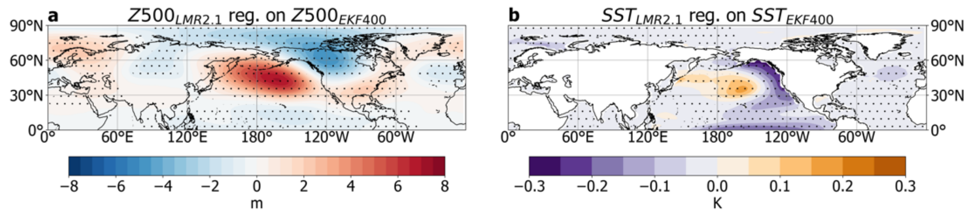

Supplementary Figure 12. Regression of the LMR2.1 (a) Z500 and (b) SST fields onto the Z500 EC(1) time-series of the MCA from EKF400. The area-weighted global mean is removed from the LMR2.1 fields before the regression. Hatching indicates statistically significant regression coefficients ($p<0.05$).
